# Preparation of Composite Nanofiber Membranes via Solution Blow Spinning and Solution Impregnation Method for CO_2_ Capture

**DOI:** 10.3390/ma18102303

**Published:** 2025-05-15

**Authors:** Kaiwen Yang, Yun Wang, Changshun Zhu, Weiguang Wu, Xuefei Fan

**Affiliations:** 1 School of Mechanical Engineering, Jiangsu University, Zhenjiang 212013, China; kaiwenyujs@163.com (K.Y.);; 2 Zhenjiang Sanwei Conveying Equipment Co., Ltd., Zhenjiang 212013, China

**Keywords:** solution blow spinning, nanofibers, carbon capture, polyamide 66, TEPA

## Abstract

Carbon dioxide (CO_2_) capture is a pivotal technology for achieving the goal of carbon neutrality. This paper proposes a novel process, SBS + SI, which integrates Solution Blow Spinning (SBS) and Solution Impregnation Method (SI), using polyamide 66 (PA66) as the carrier material and high-purity tetraethylenepentamine (TEPA) as the modifier, to fabricate nanofiber adsorption membranes with varying carrier structures and modifier component loadings. The CO_2_ adsorption performance and pore structure of the adsorbents were investigated using characterization techniques, such as Scanning Electron Microscopy (SEM), Thermogravimetric Analysis (TGA), Brunauer-Emmett-Teller (BET) surface area and pore size analysis, and Fourier Transform Infrared Spectroscopy (FT-IR). The results indicate that as the mass fraction of TEPA increases, the pores in the nanofiber membranes gradually decrease, while the CO_2_ adsorption capacity significantly increases. The PA66 nanofiber membrane achieves peak CO_2_ capture performance (44.7 mg/g at 25 °C) at 15% TEPA loading. Meanwhile, the composite nanofiber membranes also exhibit outstanding CO_2_/N_2_ selectivity with a separation factor reaching 28. Thermal regeneration tests at 90 °C confirm the composite’s outstanding cyclic stability and regenerability, demonstrating its potential for practical carbon capture applications. These findings suggest that the nanofiber adsorbents prepared by the SBS + SI process have broad application prospects in the field of CO_2_ capture.

## 1. Introduction

Carbon dioxide (CO_2_), a key greenhouse gas, is widely recognized as the main driver of global warming and climate change [1]. The combustion of fossil fuels is the leading source of excessive CO_2_ emissions. With carbon neutrality goals in mind, there is an urgent need for effective CO_2_ capture and separation technologies. Currently, pre-combustion, oxy-fuel combustion, and post-combustion capture are the primary methods. Among these, post-combustion capture has gained attention for its flexible systems, broad applicability, and compatibility with existing power plants [2]. Various separation techniques, including solid adsorption, liquid absorption, cryogenic distillation, and membrane separation, are widely used [3]. Liquid amine solutions, a common liquid absorption method, have proven effective in industrial applications but face challenges such as toxicity, corrosiveness, and high energy consumption during regeneration [4,5,6]. Thus, there is a need for new CO_2_ capture materials that are easy to process, environmentally friendly, cost-effective, and require less energy for regeneration [7,8,9].

Porous carbon-based materials have emerged as leading candidates for CO_2_ capture applications, owing to their exceptional structural tunability, high surface area, and robust chemical stability under operational conditions [10,11,12,13]. However, their inherent brittleness severely limits their practical applications. Therefore, the development of new materials with both high CO_2_ capture efficiency, good flexibility, and sufficient mechanical strength has become a key research focus. Polymer nanocomposites have emerged as a potential alternative with their unique combination of properties [14,15,16]. Zhang et al. [17] developed amine-modified porous nanofiber membranes mimicking balsam-pear skin morphology for CO_2_ capture. They prepared PAN/PVP composite fibers via electrospinning, followed by PVP removal through hydrolysis. The resulting PAN nanofibers were then grafted with polyethyleneimine (PEI), which introduced CO_2_ adsorption sites while maintaining the original porous structure. The PEI-functionalized membranes achieved a CO_2_ adsorption capacity of 1.23 mmol/g at 40 °C. Wang et al. [18] investigated the effects of nitric acid (HNO₃) and TEPA treatments on the physicochemical properties of modified and activated polyacrylonitrile (PAN)-based nanofibers. The results demonstrated that the final CO_2_ adsorption capacity was significantly enhanced, showing increases of 39% at 0.3 vol% CO_2_ concentration and 57% under pure CO_2_ conditions. Moreover, by introducing specific functional groups, the material’s selectivity and adsorption efficiency for CO_2_ can be further enhanced [19,20,21,22], opening up broader application prospects. Among various amine-based modifiers, polyethylenepolyamine compounds (e.g., DETA, TETA, and MEA) have attracted significant attention due to their abundant amine functional groups that enable efficient CO_2_ chemisorption [18,23,24]. However, these conventional amines suffer from limitations including volatility and poor thermal stability, which restrict their practical applications and lead to performance degradation after repeated adsorption-desorption cycles [25]. In contrast, tetraethylenepentamine (TEPA) demonstrates superior characteristics, maintaining high CO_2_ capture capacity even at low concentrations while exhibiting excellent adaptability across wide temperature/pressure ranges. Furthermore, TEPA-modified adsorbents can be regenerated through either thermal desorption or pressure swing processes for cyclic utilization [26].

Electrospinning is a commonly used method for preparing polymer nanofibers, but it has limitations, including a dependence on high voltage and high dielectric constant of the solution [27]. In recent years, solution blow spinning (SBS) has attracted significant attention in both academia and industry as an emerging fiber manufacturing technology due to its economic efficiency, wide applicability of raw materials, and suitability for batch production [28].

In this study, polyamide 66 (PA66) was used as the matrix material, and high-purity TEPA was used as the modifier. A novel process combining SBS and solution impregnation (SI) was proposed to prepare nanofiber membranes with various carrier structures and modification component loadings. PA66 not only exhibits excellent spinnability, mechanical properties, and thermal stability but also contains abundant repeating amide groups (CO-NH) in its molecular chain [29,30]. Due to the strong polarity of its amide groups, PA66 offers advantages over other polymers for CO_2_ capture. Using solution blow spinning, nanofiber mats with diameters smaller than 0.60 μm were fabricated. This porous structure exhibits a high surface area, superior flexibility, and moderate mechanical strength, making it highly efficient for CO_2_ adsorption. The incorporation of TEPA not only creates additional active sites for CO_2_ binding but also boosts the membrane’s selectivity and affinity toward CO_2_. Furthermore, TEPA reinforcement enhances the composite’s mechanical durability, underscoring its suitability for CO_2_ capture and other potential applications.

## 2. Materials and Methods

### 2.1. Materials and Fabrication Methods

#### 2.1.1. Materials

Polyamide 66 (PA66, viscosity: 135 g/mL) and formic acid (98%) were purchased from Shanghai Aladdin Biochemical Technology Co., Ltd. (Shanghai, China), while tetraethylenepentamine (TEPA) (99%) was obtained from Shanghai Linen Technology Development Co., Ltd. (Shanghai, China). All chemicals, including anhydrous ethanol, were used without further purification.

#### 2.1.2. Preparation of PA66-TEPA Nanofiber Membranes via SBS + SI Method

**Figure 1:**
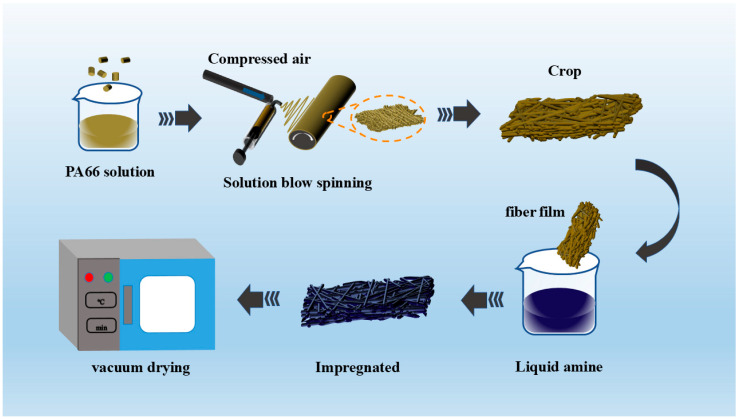
Schematic diagram of the SBS + SI preparation process of PA66-TEPA.

The fabrication of PA66-TEPA nanofiber membranes comprises two sequential steps (Figure 1): (1) solution blow spinning of PA66 fibrous scaffolds, followed by (2) surface functionalization through TEPA solution impregnation.

The preparation of PA66 nanofibers via solution blow spinning (SBS) involves dissolving 2.6 g PA66 microspheres in 10 g formic acid (26 wt%) under continuous stirring for 6 h at room temperature. The resulting homogeneous solution was loaded into a 20 mL syringe and used with an injection pump at a constant feed rate of 4.5 mL/h under 175 kPa air pressure. Nanofibers were collected on a rotating drum (250 rpm, 5 cm/s reciprocal motion) covered with tin foil, positioned 50 cm from the needle tip to facilitate fiber deposition and subsequent membrane formation

The amine impregnation method involves a solution-based process where amine compounds are adsorbed onto a support material via non-covalent interactions [31]. The amine modification of PA66 nanofiber membranes was performed through solution impregnation as follows: The thinner edges of the fiber membrane are trimmed and placed at the bottom of a beaker. A TEPA solution is prepared by mixing TEPA with anhydrous ethanol in specified proportions and stirring until homogeneous. The solution is then added to the beaker containing the fiber membrane, and the beaker is subjected to ultrasonic-assisted impregnation for 30 min. After impregnation, the composite fiber membrane is transferred to a vacuum drying oven set at 90 °C and dried for 8 h. The resulting samples are designated as PA66-TEPA-xx, where “xx” refers to the TEPA concentration in the solution. The concentrations used are 0%, 5%, 15%, and 20%, yielding samples PA66-TEPA-0, PA66-TEPA-5, PA66-TEPA-10, PA66-TEPA-15, and PA66-TEPA-20, respectively.

### 2.2. Performance Evaluation

#### 2.2.1. CO_2_ Adsorption Performance of PA66 Composite Nanofiber Membranes

The CO_2_ capture performance was assessed via TGA using 10 mg of membrane samples. Following moisture removal (N_2_ purge, 200 mL/min, 90 °C, 60 min), adsorption was measured at 25 °C under CO_2_ flow (200 mL/min) until mass stabilization. Subsequent desorption at 90 °C (N_2_ atmosphere) completed one cycle, with six repeats demonstrating membrane stability. Adsorption capacities were derived from mass differentials.

#### 2.2.2. Mechanical Properties of PA66 Composite Nanofiber Membranes

The complete membrane was removed from the aluminum foil and cut into strips with dimensions of 40 mm in length and 30 mm in width. The thickness of the membrane was measured using a micrometer, and the average value was taken. The tensile properties, including the breaking stress and breaking elongation, of the composite nanofiber membrane were tested using a tensile testing machine (PT-305; Guangdong Beidou Precision Instrument Corporation; Dongwan, China) under the following conditions: a clamp distance of 30 mm and a testing speed of 5 mm/min. The breaking stress and breaking elongation were calculated as follows:

(1) $$\sigma =\frac{P}{S}$$

(2) $$\varepsilon =\frac{L-L_{0}}{L_{0}}$$

where **σ** is the breaking stress (N/m^2^), **P** is the breaking force (N), **S** is the cross-sectional area (m^2^), **ε** is the breaking elongation (%), **L₀** is the length of the fiber after applying pre-tension and straightening (mm), and **L** is the length of the fiber at the point of fracture (mm).

#### 2.2.3. Calculation of CO_2_/N_2_ Adsorption Selectivity

It is essential to analyze the CO_2_/N_2_ selectivity of the synthesized samples to evaluate the feasibility of CO_2_ capture under practical conditions. Adsorption selectivity is a crucial characteristic of an adsorbent, equally important as its adsorption capacity. High selectivity indicates the ability of the adsorbent to effectively distinguish CO_2_ from other coexisting gases, thereby enhancing separation efficiency.

The CO_2_ and N_2_ adsorption isotherms were fitted with a virial-type equation to calculate the Henry’s law selectivity of CO_2_ over N_2_ at 25 °C for PA66-TEPA [32,33]:

(3) $$\ln\left(n/p\right)=A_{0}+A_{1}n+A_{2}n^{2}+\cdots$$

where *p* (Pa) is the pressure and *n* (mol/g) is the gas adsorbed amount, *Ai* (*i* = 1, 2, 3 ···) is a virial coefficient. The first virial coefficient (*A*_0_) is associated with the energy of adsorbate-adsorbent interactions, while the second (*A*_1_) is associated with the energy of adsorbate-adsorbate interactions. At low surface coverage, *Ai* (*i* = 2, 3, 4 ···) could be negligible, allowing the equation to be reduced to:

(4) $$\ln\left(n/p\right)=A_{0}+A_{1}n$$

At low surface coverage, ln(*n*/*p*) shows a linear dependence on the adsorbed amount *n*. The values of *A*_0_ and *A*_1_ could thus be obtained from the slope and the intercept. The Henry’s constant (K_H_) can be extracted from the value of the first coefficient using the following expression:

(5) $$K_{H}=\exp\left(A_{0}\right)$$

The Henry’s law selectivity for CO_2_ over N_2_ can be determined based on the ratio of their Henry’s constant $K_{H\left({CO}_{2}\right)}$ and $K_{H\left(N_{2}\right)}$, respectively:

(6) $$S=\frac{K_{H\left({CO}_{2}\right)}}{K_{H\left(N_{2}\right)}}$$

By applying this approach, the selective adsorption performance of the PA66-TEPA composite membrane toward CO_2_ can be accurately assessed, providing valuable insights into its potential application in industrial flue gas separation.

### 2.3. Characterization

The morphology of PA66 nanofibers and PA66-TEPA nanocomposite membranes was examined using scanning electron microscopy (SEM5000, Guoyi Quantum Technology Corporation, Hefei, China). The samples were sputter-coated with gold before imaging to enhance fiber conductivity. Fourier transform infrared spectroscopy (VERTE70, Bruker Corporation, Berlin, Germany) was used to analyze the materials’ functional groups. The membranes were characterized for their physicochemical properties using multiple analytical techniques. Surface area and pore structure analysis were conducted via nitrogen physisorption measurements (ASAP 2020 PLUS, Micromeritics Instrument Corporation, Norcross, GA, USA) based on the Brunauer-Emmett-Teller (BET) method. Thermal stability and CO_2_ adsorption performance were evaluated by thermogravimetric analysis (TGA-550, TA Instruments, New Castle, DE, USA) under controlled atmospheric conditions.

## 3. Results

### 3.1. Morphological Characterization of Composite Nanofiber Membranes

**Figure 2:**
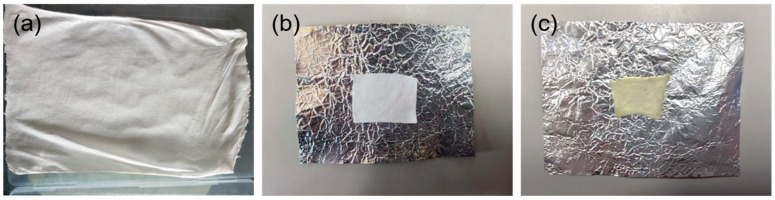
Fabrication process stages: (**a**) Initial collection, (**b**) After cropping, and (**c**) impregnation.

Figure 2 displays the images of three key stages in the fabrication process of the PA66-TEPA composite nanofiber membrane. Figure 2a shows the pristine PA66 nanofiber membrane prepared via solution blow spinning. The membrane exhibits a uniform fibrous network structure with a smooth surface, demonstrating excellent film-forming properties. Figure 2b presents the trimmed pristine PA66 nanofiber membrane, where the thinner edges were removed. The cropped membrane has neat edges, facilitating subsequent experimental procedures. The trimming process did not significantly affect the microstructure, and the fibrous network remained intact. Figure 2c illustrates the PA66-TEPA composite nanofiber membrane after liquid amine impregnation. Through these three consecutive processing stages, a functionalized PA66-TEPA composite nanofiber membrane was successfully prepared, laying a solid foundation for further performance testing and application research.

**Figure 3:**
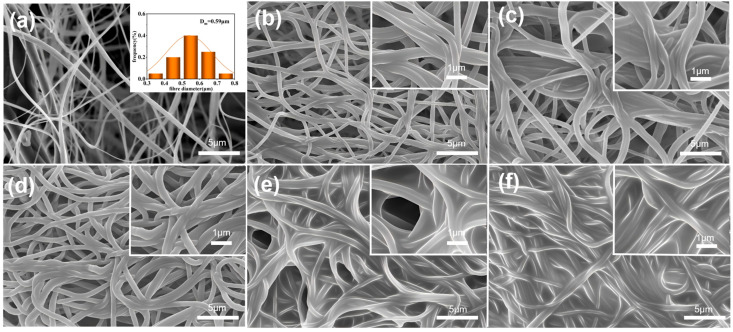
The SEM images of composite fiber membranes modified with varying TEPA concentrations: (**a**) PA66, (**b**) PA66-TEPA-0, (**c**) PA66-TEPA-5, (**d**) PA66-TEPA-10, (**e**) PA66-TEPA-15, and (**f**) PA66-TEPA-20.

Figure 3 presents the morphological evolution of composite membranes with varying TEPA concentrations. The unmodified PA66 nanofibers (Figure 3a), fabricated by solution blow spinning (SBS), display smooth surfaces and uniform diameters (590 nm), forming an interconnected porous network. This pristine fibrous architecture provides an ideal substrate for subsequent amine functionalization. The pore size and structure of porous carriers play a selective role in CO_2_ adsorption, enabling preferential adsorption of smaller CO_2_ molecules while exhibiting weaker adsorption capacity for larger molecules, such as nitrogen, thus further enhancing the selectivity for CO_2_ [34]. Figure 3b–f show the morphology of PA66-TEPA-0 to PA66-TEPA-20. It can be observed that as the PA66 nanofiber membrane, prepared by SBS, is impregnated with TEPA solutions of varying concentrations, the average fiber diameter increases significantly with higher TEPA concentration (Figure 3b–f), indicating successful deposition of TEPA onto the membrane. Figure 3c,d clearly show that as the TEPA concentration rises, the TEPA layer thickens, covering most fibers and forming adhesive structures between adjacent fibers. When the TEPA concentration exceeds a critical threshold, a much thicker polymer membrane forms, covering most of the fiber surface and blocking the pores both between and inside the fibers. At a concentration of 20 wt%, the pores between the nanofibers are nearly completely filled with polymer, resulting in a continuous membrane (Figure 3f), primarily due to the high molecular weight of the TEPA solution.

### 3.2. Molecular Structure Analysis of Composite Nanofiber Membranes

**Figure 4:**
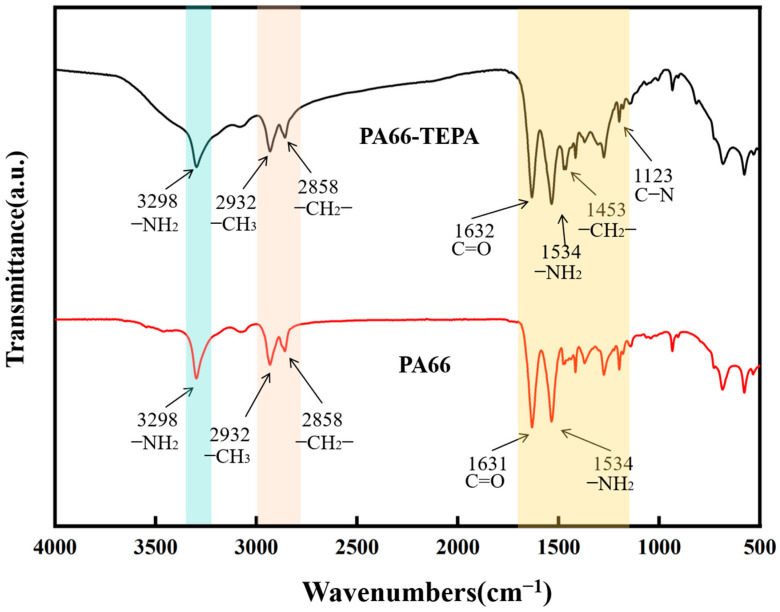
FT-IR spectra of PA66 and PA66-TEPA composite nanofiber membranes.

Figure 4 shows the FTIR spectra of the original PA66 membrane and the TEPA-impregnated membrane. It can be observed that both membranes exhibit similar spectra, with distinct absorption bands corresponding to the methylene and amide groups. The infrared characteristic peaks of PA66’s amide linkages (-CO-NH-) are as follows: 3298 cm⁻^1^ corresponds to the N-H stretching vibration of the amine group (-NH_2_); 1631 cm⁻^1^ is the main characteristic absorption peak of the amide I band, corresponding to the C=O stretching vibration of the carbonyl group in the amide; and 1534 cm⁻^1^ corresponds to the N-H bending vibration of the amine group (-NH_2_) in the amide. The methylene (-CH_2_-) groups’ peaks are as follows: 2932 cm⁻^1^ corresponds to the C-H stretching vibration of the methyl group (-CH₃), and 2858 cm⁻^1^ corresponds to the C-H stretching vibration of the methylene group (-CH_2_-) [35]. For TEPA, characteristic peaks appear at 1453 cm⁻^1^ and 1123 cm⁻^1^, with the former corresponding to the C-H bending vibration of the methylene group (-CH_2_-) and the latter corresponding to the C-N bending vibration in the TEPA molecule [36,37]. Additionally, the peak changes in the 1300–1700 cm⁻^1^ range further confirm the successful loading of TEPA onto the membrane.

### 3.3. Pore Structure Analysis of Composite Nanofiber Membranes

**Figure 5:**
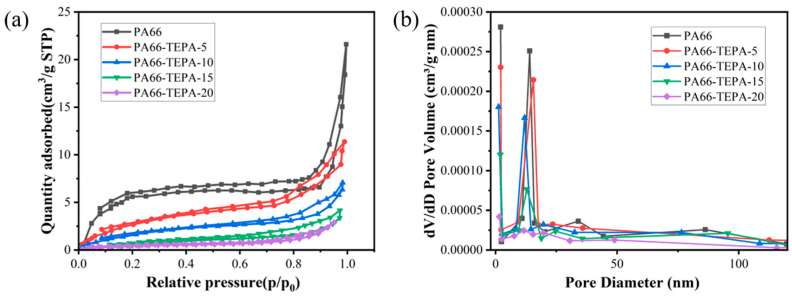
(**a**) N_2_ adsorption/desorption isotherms and (**b**) pore size distribution of the composite nanofiber membranes.

Porosity plays a crucial role in determining the CO_2_ adsorption efficiency of membranes. The pore structure of the PA66-TEPA composite nanofiber membranes was characterized using the Brunauer–Emmett–Teller (BET) method at −196 °C. The nitrogen adsorption/desorption isotherms obtained can be used to analyze the porosity and structural characteristics of the composite nanofiber membranes, as shown in Figure 5a. All nanofiber membranes exhibit typical Type IV isotherms [38], indicating the presence of mesopores, and the composite nanofiber membranes retain a porous fiber structure after TEPA treatment. The adsorption curve of the original PA66 nanofiber membrane shows a rapid increase in nitrogen adsorption at relatively low pressures (0–0.1 p/p₀), corresponding to monolayer adsorption, which suggests the presence of micropores. Subsequently, the adsorption increases slowly until 0.9 p/p₀, corresponding to multilayer adsorption. Finally, at higher relative pressures (0.9–1 p/p₀), the adsorption increases sharply due to capillary condensation, indicating the presence of mesopores. By observing the nitrogen adsorption/desorption isotherms of PA66-TEPA-5, PA66-TEPA-10, PA66-TEPA-15, and PA66-TEPA-20, it can be seen that the composite nanofiber membranes and the original PA66 nanofiber membrane exhibit similar isotherms, with the presence of mesopores, indicating the potential for CO_2_ adsorption applications. However, compared to the original PA66 nanofiber membrane, the isotherms of the composite nanofiber membranes show lower initial nitrogen adsorption, and the isotherms at higher relative pressures also exhibit smaller slopes. This suggests that as the TEPA concentration increases, the pore sizes of the micropores and mesopores in the composite nanofiber membranes decrease, which is also evident in the pore size distribution plot and SEM images shown in Figure 3. Additionally, the presence of TEPA blocks the pores between and inside the fibers, leading to a significant reduction in specific surface area and total pore volume (Table 1). The specific surface areas of pure PA66, PA66-TEPA-5, PA66-TEPA-10, PA66-TEPA-15, and PA66-TEPA-20 were 17.31 m^2^/g, 7.51 m^2^/g, 5.21 m^2^/g, 3.75 m^2^/g, and 1.56 m^2^/g, respectively. It can be observed that the original PA66 nanofiber membrane has the highest BET surface area, and the unobstructed pores between and inside the fibers ensure a higher BET surface area. Figure 5b presents the Barrett-Joyner-Halenda (BJH) plot, which is used to analyze the pore structure, including pore size and volume. It is clear from the plot that the original PA66 nanofibers contain a notable amount of micropores and mesopores, with a pore volume of 0.23 cm^3^/g. The pore volume of the composite nanofiber membranes decreases with increasing TEPA impregnation concentration. Since the specific surface area, pore size, and TEPA loading are key factors influencing the CO_2_ adsorption performance of the composite membrane, optimal CO_2_ adsorption can only be achieved when these factors are balanced. These results demonstrate that the successful incorporation of TEPA, as confirmed by FTIR, directly influences the membrane’s porous structure. The BET measurements show a clear trend of decreasing specific surface area and pore volume with increasing TEPA loading, which is attributed to physical occupation of pores by TEPA molecules. The agreement between these characterization techniques confirms the controlled modification of membrane properties through TEPA incorporation.

### 3.4. Mechanical Properties of Composite Nanofiber Membranes

**Figure 6:**
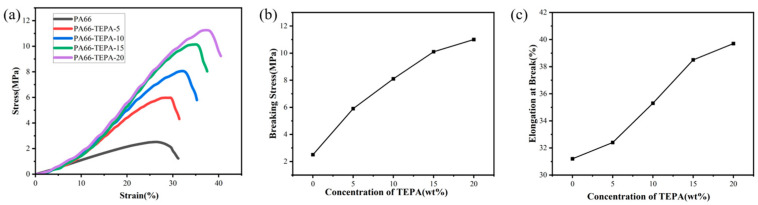
Stress-strain characteristics of composite nanofiber membranes with different TEPA impregnation concentrations: (**a**) Stress-strain curves, (**b**) Fracture elongation, and (**c**) Fracture stress.

The mechanical properties of the composite nanofiber membranes obtained with different TEPA impregnation concentrations are shown in Figure 6. Compared with the original PA66 membrane, the synthesized composite nanofiber membranes exhibit excellent flexibility and enhanced mechanical properties. Figure 6a presents the stress-strain characteristics of pure PA66 membranes and composite nanofiber membranes with varying TEPA impregnation concentrations. It can be observed that the flexibility and stress strength of the composite nanofiber membranes impregnated with TEPA are significantly improved compared to the pure PA66 nanofiber membrane. As the TEPA concentration increases, the mechanical strength of the material increases significantly from 2.5 MPa to 11.3 MPa, which is attributed to the successful attachment of TEPA to the original PA66 nanofiber membrane, with the thickness of the attached layer increasing as the TEPA impregnation concentration rises.

Figure 6b,c show that as the TEPA impregnation concentration increases from 0 wt% to 15 wt%, the fracture stress of the composite nanofiber membranes exhibits a steady increase. However, when the concentration increases from 15 wt% to 20 wt%, the rate of increase in fracture stress slows down, and a similar trend is observed in the fracture elongation. This behavior can be attributed to the TEPA layer, which strengthens the cross-linking force between the nanofibers attached to the original PA66 membrane [39]. As a result, the diameter of individual nanofibers increases, and thus, when a single nanofiber breaks, it has to withstand a larger tensile load, thereby improving both the fracture stress and fracture elongation of the composite nanofiber membranes.

### 3.5. CO_2_ Adsorption Performance of Composite Nanofiber Membranes

**Figure 7:**
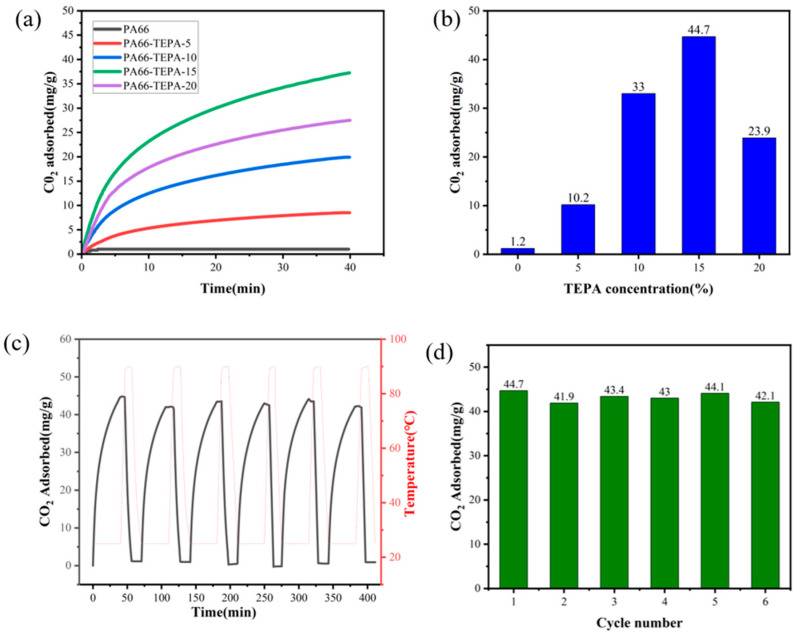
(**a**) CO_2_ adsorption isotherms of nanofiber membranes at 25 °C; (**b**) Effect of TEPA concentration on CO_2_ adsorption; (**c**) CO_2_ adsorption/desorption cycles; (**d**) Cycling CO_2_ adsorption capacity of composite nanofiber membranes.

Figure 7a presents the CO_2_ adsorption performance of pure PA66 and TEPA-impregnated composite nanofiber membranes, as tested by thermogravimetric analysis (TGA). It can be observed that the original PA66 nanofiber membrane exhibits almost negligible CO_2_ adsorption. However, after impregnation with TEPA solution, the CO_2_ adsorption capacity of the composite nanofiber membrane significantly increased, with adsorption amounts ranging from 10 to 44.7 mg/g. This enhancement is attributed to the abundant amine groups (-NH_2_) present in TEPA, which can react with CO_2_ in a dry environment to form carbamate salts, thereby increasing the CO_2_ adsorption.

Figure 7b demonstrates the effect of different TEPA concentrations on CO_2_ adsorption. As the TEPA concentration increases, the CO_2_ adsorption capacity of the composite nanofiber membranes steadily improves. However, when the TEPA concentration reaches 20%, the CO_2_ adsorption performance declines by 20.8 mg/g. This decrease is attributed to the complete blockage of the fiber pores when the TEPA concentration reaches 20 wt%, thereby decreasing the available space for CO_2_ adsorption in the fibers [40]. This is also confirmed by the scanning electron microscope (SEM) images (Figure 3c–f), which show the pore-blocking effect.

Further studies were conducted on the optimized TEPA-impregnated composite membranes to assess the adsorption stability of the composite nanofiber membranes after multiple cycles. The CO_2_ adsorption performance was evaluated through multiple adsorption cycles, with the results shown in Figure 7c. Throughout the testing process, the adsorption capacity of the composite nanofiber membrane showed only slight fluctuations, confirming the stable cycling performance of the TEPA-impregnated PA66 nanofiber composite membrane. Figure 7d displays the adsorption amount in each cycle. The cyclic adsorption-desorption tests revealed excellent recyclability of the PA66-TEPA membranes. While the first cycle showed the highest CO_2_ uptake, subsequent cycles exhibited a marginal decrease (~2.6 mg/g after 6 cycles), retaining 94% of the initial capacity (Figure 7d). This minor reduction likely stems from residual CO_2_ remaining after the 10-min desorption phase (90 °C, N_2_), suggesting that slightly longer regeneration times or higher temperatures may further improve reversibility. This demonstrates exceptional regenerability for practical carbon capture applications. The consistent cycle-to-cycle reproducibility further confirms the structural integrity and amine stability of the PA66-TEPA composite under operational conditions.

Table 2 presents the adsorption temperatures and capacities of some previously reported adsorbents. It can be seen that PA66-TEPA exhibits a good adsorption capacity at room temperature (25 °C), and due to the excellent flexibility and mechanical properties of the PA66 nanofiber matrix, it can be applied in a wider range of environments.

### 3.6. Selective CO_2_/N_2_ Adsorption Performance of Composite Nanofiber Membranes

Moreover, an excellent CO_2_ capture material must not only exhibit high and stable CO_2_ adsorption capacity with superior regeneration performance but also demonstrate high selectivity for CO_2_ molecules in flue gas. Since CO_2_ and N_2_ are the two primary components of flue gas, where CO_2_ accounts for only 12–15% while N_2_ makes up 70–75%, the designed adsorbent must possess a strong affinity for CO_2_ in a mixed gas environment to achieve effective separation.

**Figure 8:**
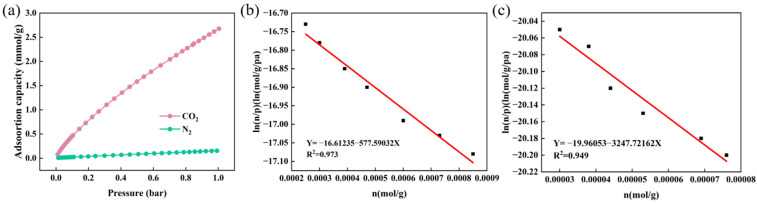
(**a**) CO_2_ and N_2_ adsorption isotherms at 25 °C; The virial characteristic curves of (**b**) CO_2_ and (**c**) N_2_ for PA66-TEPA-15 at 0 °C.

Figure 8a presents the CO_2_ and N_2_ adsorption isotherms of the optimized composite nanofiber membrane PA66-TEPA-15 at 25 °C. At 1 bar and 25 °C, PA66-TEPA-15 exhibits a CO_2_ uptake of 2.67 mmol/g, significantly higher than its N_2_ adsorption capacity (0.15 mmol/g). The adsorption isotherms of CO_2_ and N_2_ were fitted using Henry’s law to evaluate the CO_2_/N_2_ selectivity of the composite nanofibers [33,47]. Figure 8b,c display the Henry’s law characteristic curves for CO_2_ and N_2_ adsorption on PA66-TEPA-15, while Table 3 summarizes the corresponding Henry’s constants and fitting results. As calculated from the ratio of their Henry’s constants using Equation (6), the Henry’s law selectivity (S) for CO_2_ over N_2_ reached 28, demonstrating its outstanding CO_2_/N_2_ selective adsorption capability. These findings highlight the great potential of the PA66-TEPA composite membrane for efficient CO_2_ separation in industrial flue gas applications.

### 3.7. CO_2_ Adsorption Mechanism

The adsorption mechanism of CO_2_ in different materials can generally be classified into two categories: physisorption and chemisorption. Physisorption is governed by van der Waals forces, where CO_2_ molecules are adsorbed onto the porous structure of the adsorbent with relatively weak interactions [48]. In contrast, chemisorption involves the formation of chemical bonds between CO_2_ molecules and active functional groups, significantly enhancing the binding strength and improving CO_2_ capture efficiency [49].

The CO_2_ saturation adsorption capacity of porous materials is primarily determined by two key parameters: the number of active adsorption sites and the structural characteristics of the material’s porosity. The PA66-TEPA composite nanofiber membrane, developed in this study, exhibits enhanced CO_2_ adsorption performance due to the synergistic effect between the porous PA66 nanofiber structure and the amine functional groups in TEPA.

Experimental results indicate that when CO_2_ molecules reach the modified composite membrane’s surface, they are effectively trapped within the membrane via a combination of physisorption on the PA66 nanofiber framework and chemisorption by TEPA. The hierarchical porous structure of the PA66 nanofiber framework provides efficient transport pathways for CO_2_ molecules, while the amine groups in TEPA react with CO_2_ to form stable carbamate structures through neutralization reactions [23], as described below:

(7) $$CO_{2}+2RNH_{2}⇄RNHCOO^{-}+RNH_{3}^{+}$$

(8) $${CO}_{2}+2R_{2}NH⇄R_{2}NH_{2}^{+}+R_{2}NCOO^{-}$$

This coupled mechanism of physisorption and chemisorption significantly enhances the CO_2_ adsorption capacity of PA66-TEPA. In the adsorption process, most of the amine groups originate from TEPA, which contains two primary amines and three secondary amines, enabling 1 mol of CO_2_ to react with 2 mol of TEPA. Once the reaction reaches completion, CO_2_ molecules diffuse into the composite membrane’s porous network until saturation is achieved. These findings confirm that the number of active functional sites and the porous structure play a crucial role in regulating the CO_2_ adsorption capacity of the composite membrane. The CO_2_ adsorption process of the amine-modified PA66-TEPA composite nanofiber membrane is exothermic and reversible. Therefore, increasing temperature reverses the reaction and reduces adsorption capacity by promoting the decomposition of carbamate (RNHCOO⁻) formed between amine groups and CO_2_, thereby releasing CO_2_ and regenerating amine groups [50,51,52]. As the temperature rises, the average kinetic energy of CO_2_ gas molecules increases significantly, leading to more vigorous molecular motion. When the molecular kinetic energy exceeds the van der Waals binding energy on the material surface, adsorbed CO_2_ molecules detach from the surface. Moreover, elevated temperatures may cause microstructural changes in the PA66-TEPA composite, such as pore structure deformation or reduced activity of adsorption sites [53], which further diminishes the material’s CO_2_ adsorption capacity at high temperatures.

## 4. Conclusions

In this work, an SBS + SI method was proposed to successfully anchor TEPA onto the surface of PA66 nanofiber membranes, resulting in the preparation of PA66-TEPA composite nanofiber membranes. The pore structure of the membranes could be effectively tuned by adjusting the TEPA impregnation concentration. It was found that a 15 wt% TEPA impregnation concentration provided the optimal CO_2_ adsorption performance, with an impressive tensile strength of up to 10.1 MPa. The PA66-TEPA exhibited a BET surface area of 3.75 m^2^/g and a rich porous structure, with a significant amount of mesopores. Meanwhile, the composite nanofiber membranes also exhibit outstanding CO_2_/N_2_ selectivity with a separation factor reaching 28. After TEPA impregnation, the composite membrane was able to adsorb up to 44.7 mg of CO_2_ per gram. Furthermore, the membrane demonstrated excellent performance stability, as evidenced by the CO_2_ adsorption/desorption cycle test, which showed that it could be reused multiple times as a CO_2_ adsorbent.

Throughout the adsorption process, the introduction of TEPA played a critical role in enhancing the adsorption performance. On the one hand, TEPA improved the flexibility and tensile strength of the nanofiber membrane, enabling its application in a broader range of environments. On the other hand, TEPA provided a substantial number of amine groups, creating additional CO_2_ capture sites and enhancing the membrane’s affinity and selectivity toward CO_2_. This makes the PA66-TEPA composite membrane a promising candidate for CO_2_ capture and related applications.

## Figures and Tables

**Table 1:** Pore volumes, average pore sizes, and specific surface areas (S_BET_) of PA66, PA66-TEPA-5, PA66-TEPA-10, and PA66-TEPA-20.

Sample	Pore Volume (cm^3^/g)	S_BET_ (m^2^/g)
PA66	0.23	17.31
PA66-TEPA-5	0.18	7.51
PA66-TEPA-10	0.15	5.21
PA66-TEPA-15	0.11	3.75
PA66-TEPA-20	0.02	1.56

**Table 2:** Comparison of our optimized sample’s CO_2_ adsorption capacity with the literature-reported sorbents.

Sample	Adsorption Temperature (°C)	CO_2_ Adsorption Capacity (mg g^−1^)	Ref.
Mesoporous TiO_2_ bead bentonite	30	18	[41]
Porous organic polymer	25	42	[42]
PA66-TEPA	25	44.7	This work
Commercial activated carbon	25	49	[31]
NiCoAl-LDO	0	55	[43]
COF-300-NO_2_	25 (100 kPa)	187	[44]
COF-300-SO_3_H	25 (100 kPa)	274	[44]
C_4_-IL/ICOF	0	71	[45]
HCP1	0	64.1	[46]

**Table 3:** Henry’s constants and virial fitting results for PA66-TEPA-15 at 25 °C.

Sample	Gas, Temperature (°C)	K_H_	R^2^
PA66-TEPA-15	CO_2_, 25	6.1 × 10^−8^	0.973
PA66-TEPA-15	N_2_, 25	2.14 × 10^−9^	0.946

## Data Availability

The original contributions presented in this study are included in the article. Further inquiries can be directed to the corresponding author.
